# The Incremental Role of Stress Echocardiography in Valvular Heart Disease: A Narrative Review

**DOI:** 10.3390/diagnostics16010148

**Published:** 2026-01-02

**Authors:** Adriana Correra, Alfredo Mauriello, Carmen Del Giudice, Celeste Fonderico, Matilde Di Peppo, Vincenzo Russo, Antonello D’Andrea, Giovanni Esposito, Natale Daniele Brunetti

**Affiliations:** 1 Cardiology Department, Ospedali Riuniti University Hospital, Viale Pinto 1, 71122 Foggia, Italy; matilde.dipeppo@gmail.com (M.D.P.); natale.brunetti@unifg.it (N.D.B.); 2 S.C. Cardiology, Institute National Cancer, IRCCS, Foundation “G. Pascale”, Via M. Semmola 52, 80131 Naples, Italy; alfredo.mauriello93@libero.it (A.M.); celeste.fonderico@istitutotumori.na.it (C.F.); 3 Cardiology Unit, Boscotrecase Hospital, ASL NA3Sud, Via Lenza, 3, 80042 Boscotrecase, Italy; carmen.delgiu93@gmail.com; 4 Cardiology Unit, Department of Medical and Translational Sciences, University of Campania “Luigi Vanvitelli”, “V. Monaldi” Hospital, Via Leonardo Bianchi snc, 80131 Naples, Italy; vincenzo.russo@unicampania.it; 5 Cardiology and Intensive Care Unit, Department of Cardiology, “Umberto I” Hospital, Via Alfonso De Nicola 1, 84014 Nocera Inferiore, Italy; antonellodandrea@libero.it; 6 Cardiology Unit, Department of Advanced Biomedical Sciences, University of Naples Federico II, Via S. Pansini 5, 80131 Naples, Italy; giovanni.esposito2@unina.it

**Keywords:** echocardiography, valvular heart disease, mitral, aortic, stress echocardiography

## Abstract

**Background/Objectives:** The diagnosis and risk stratification of valvular heart disease have traditionally relied on resting echocardiography. However, in a significant portion of patients, resting findings do not fully reflect the hemodynamic severity of the condition, particularly in asymptomatic individuals with severe valvular disease or those with nonspecific symptoms. In this context, stress echocardiography emerges as a vital imaging modality, providing a dynamic assessment of valvular, ventricular, and pulmonary function under hemodynamic load (from physical exercise or pharmacological agents). **Methods:** We conducted a comprehensive synthesis and critical evaluation of the current landscape, recent advancements, and future directions regarding the application of stress echocardiography in valvular heart disease. **Results:** This comprehensive review explores the incremental role of stress echocardiography in valvular heart disease, analyzing the evolution of its clinical applications, from low-flow, low-gradient aortic stenosis to the evaluation of contractile reserve and exercise-induced pulmonary hypertension in mitral stenosis and regurgitation. We discuss standardized protocols, key parameters to monitor, and the diagnostic and prognostic outcomes from major clinical trials and current guidelines. Attention is given to stress echocardiography’s ability to unmask the true severity of the disease and to identify patients at high risk for adverse events, thereby guiding crucial clinical decisions, such as the optimal timing for surgical or transcatheter intervention. **Conclusions:** The review evaluates the limitations of modality and outlines future research directions, including its integration with new technologies like 3D echocardiography and speckle tracking techniques, to further optimize the role of stress echocardiography as a decision-making tool in the multidisciplinary management of valvular heart disease.

## 1. Introduction

Initially developed for the diagnosis and risk stratification of ischemic heart disease through the evaluation of wall motion abnormalities, stress echocardiography (SE) has expanded significantly in scope [1]. It is now increasingly utilized to assess hemodynamic responses under stress across various cardiac conditions. In particular, the evidence base for SE in valvular heart disease (VHD) has grown rapidly; consequently, current clinical guidelines now advocate for its use when making management decisions for aortic stenosis (AS) and mitral regurgitation (MR) [2]. Furthermore, research continues to yield novel insights into the application of SE for other types of valvular pathologies.

VHD remains a significant global health challenge. As the epidemiological landscape shifts from rheumatic to degenerative etiologies, the prevalence of VHD continues to rise alongside the aging populations of developed nations [3]. Over the last few decades, therapeutic options have expanded considerably due to advancements in both surgical and transcatheter interventions [3]. As the average age of patients undergoing these procedures increases, the need for precise disease severity assessment and robust risk stratification becomes even more critical. While imaging modalities such as three-dimensional echocardiography, computed tomography (CT), and magnetic resonance imaging (MRI) have been revolutionized by sophisticated scanners and quantification software, these tools primarily provide anatomical and functional data at rest. Consequently, they offer only limited insight into the complex hemodynamic shifts that occur under stress conditions. This review aims to summarize current knowledge on SE for VHD and discuss future perspectives.

## 2. Materials and Methods

This narrative review offers a comprehensive synthesis and critical evaluation of the current landscape, recent advancements, and future directions regarding the application of SE in VHD. Narrative reviews serve as an essential tool for consolidating extensive and rapidly advancing fields in cardiovascular imaging. To ensure a thorough identification of relevant literature, a search was conducted across major biomedical databases, focusing on the core concepts of the title. The search strategy employed Boolean operators (“AND”, “OR”) to combine key terms, including [4]:

Valvular Heart Disease: “Aortic stenosis” (AS), “aortic Regurgitation” (AR), “Mitral stenosis” (MS), and “Mitral regurgitation” (MR).

Clinical Evaluation: “Diagnosis,” “Biomarkers,” “Stress Echocardiography” (SE), “Dobutamine Stress Echocardiography” (DSE), and “Treatment.”

The selection criteria focused on English-language publications, encompassing literature reviews, retrospective and prospective studies, and expert editorials addressing the diagnostic and prognostic role of SE in VHD published in PubMed/MEDLINE (https://pubmed.ncbi.nlm.nih.gov/) and EMBASE (https://embase.com) from January 2000 to December 2025. While this approach does not adhere to the strict replicability requirements of a systematic review, it provides an evidence-based framework that captures the most significant clinical and scientific breakthroughs in the field.

## 3. Protocol Regarding Stress Echocardiography

SE is a highly versatile diagnostic technique that enables a dynamic assessment of myocardial structure and function under physiological or pharmacological stress conditions [5]. This method unmasks functional or hemodynamic anomalies that remain occult at rest, integrating the onset of symptoms with actual cardiac involvement [6]. SE can be performed through either physical exertion or pharmacological induction [7]. Exercise is generally the preferred modality as it maintains the body’s integrated electromechanical response and offers vital insights into a patient’s functional limits. Conversely, pharmacological agents fail to replicate the intricate hemodynamic and neurohormonal changes triggered by exercise—such as pulmonary, circulatory, and skeletal muscle responses, or psychological drive. Consequently, this review focuses specifically on exercise SE and DSE [1]. General protocols for performing an echo stress test are primarily divided into two categories: exercise stress and pharmacological stress.

### 3.1. Exercise Stress

Exercise represents the test of choice for most clinical applications, as it preserves the integrity of the natural electromechanical response and provides valuable information on the patient’s functional capacity [8]. This can be performed using a treadmill or a cycle ergometer:

- Treadmill (e.g., Bruce protocol): echocardiographic imaging is usually acquired immediately after the cessation of effort. It is imperative to complete measurements within 1–2 min before hemodynamic parameters return to baseline values [9].
- Semi-supine cycle ergometer: this offers the technical advantage of acquiring images and Doppler data during various levels of increasing workload, allowing for continuous monitoring of the cardiac response to exertion [9].

### 3.2. Pharmacological Stress

Pharmacological stress is used as an alternative when the patient has physical limitations that prevent adequate exercise.

- Dobutamine: the most common agent, an inotrope that acts on beta-1 adrenergic receptors to increase heart rate and myocardial contractility. Protocols typically involve an initial low-dose infusion (5 µg/kg/min), gradually increased every 5–8 min up to doses of 20–40 µg/kg/min to recruit contractile reserve [8]. Should the target heart rate remain unachieved at the peak dobutamine dose, supplemental atropine (0.25–0.5 mg boluses; max 2.0 mg) is provided. Beta-blockers are subsequently administered for pharmacological reversal [10].
- Vasodilators (Dipyridamole or Adenosine): these are primarily employed for the assessment of coronary flow reserve [11,12]. In the event of persistent side effects or severe ischemia during vasodilator stress, intravenous aminophylline (50–250 mg) was administered as a specific competitive antagonist of adenosine receptors.

### 3.3. Monitoring and Endpoints

During any echo stress protocol, constant monitoring of blood pressure, the electrocardiogram (ECG), and clinical symptoms is essential. The test continues until specific diagnostic endpoints are reached, such as:

- Achievement of 85% of the age-predicted maximum heart rate.
- Completion of the established workload.
- Appearance of intolerable symptoms.
- Evidence of clear echocardiographic or electrocardiographic positivity.

Despite the development of advanced imaging modalities such as MRI and CT for evaluating VHD, SE remains a cornerstone in the assessment of these conditions [13,14]. While stress MRI and stress CT represent a significant step forward in evaluating patients with VHD who are asymptomatic at baseline, they possess several limitations. These include susceptibility to arrhythmia artifacts, acceleration methods that can reduce temporal resolution—potentially leading to an overestimation of left ventricular (LV) end-systolic volume—and challenges posed by patient factors such as claustrophobia and metallic implants [15].

Furthermore, SE provides added value in evaluating hemodynamic parameters, transvalvular pressure gradients, and additional functional information, such as pulmonary artery pressure. In this context, physical exercise SE offers distinct advantages over the pharmacological stress primarily used in CT and MRI, as it accounts for venous return, pulmonary functional capacity, and peripheral muscular contribution [13,15,16].

## 4. Role of Stress Echocardiography in Valvular Heart Disease

### 4.1. Mitral Regurgitation

MR—particularly functional/secondary MR—is intrinsically dynamic and sensitive to preload, afterload, and LV contractility [1]. SE is therefore most informative when there is discordance between symptoms and resting MR severity, when MR is expected to worsen with exertion, or when the mechanism of exertional dyspnoea remains uncertain [2,17]. Importantly, MR assessment during stress should not be reduced to “re-grading MR at peak exercise”, but should integrate MR dynamics, pulmonary pressure response, and ventricular reserve (LV ± right ventricle [RV]), as these parameters are closely linked to symptoms, functional limitation, and prognosis [17,18]. Table 1 summarizes the recommended stress modality, minimum dataset, high-risk stress findings, and typical clinical implications across MR phenotypes.

#### 4.1.1. Primary or Degenerative Mitral Regurgitation

**Figure 1:**
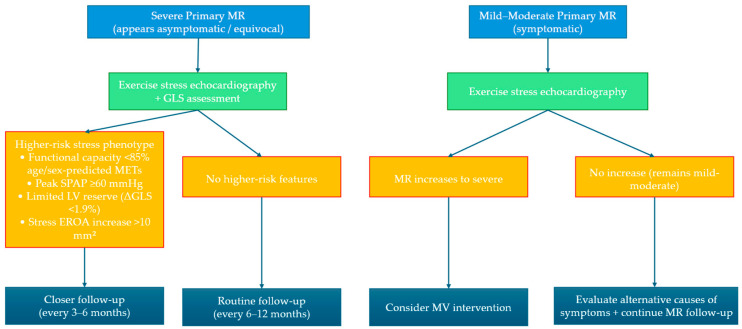
Practical workflow for exercise stress echocardiography in primary mitral regurgitation. The algorithm summarizes a structured approach to stress echocardiography according to two different clinical scenarios. GLS: global longitudinal strain; LV: left ventricle; METs: metabolic equivalent of task; MR: mitral regurgitation; MV: mitral valve; SPAP: systolic pulmonary artery pressure.

In degenerative MR, exercise SE is primarily used to unmask symptoms in apparently asymptomatic patients and refine risk stratification [17]. Stress features consistently associated with worse outcomes include (i) development of symptoms, (ii) exercise-induced pulmonary hypertension (e.g., systolic pulmonary artery pressure (SPAP) ≥ 60 mmHg), and (iii) evidence of limited LV contractile reserve, defined by a blunted rise in left ventricle ejection fraction (LVEF) and/or impaired longitudinal functional response [18,19]. When feasible, RV recruitment adds complementary prognostic information; for example, reduced exercise tricuspid anular plane systolic excursion (TAPSE) (e.g., <17 mm) has been associated with incremental risk in selected cohorts [20]. When MR is already severe at rest, re-quantification of MR severity during exercise is usually not required; imaging should prioritise pulmonary pressure dynamics and LV/RV reserve [2,21,22,23]. Figure 1 summarizes a structured approach to stress echocardiography according to two different clinical scenarios.

#### 4.1.2. Secondary or Functional Mitral Regurgitation

In secondary MR, SE is particularly useful when symptoms appear disproportionate to resting LV dysfunction or MR severity, in patients with moderate MR scheduled for coronary bypass surgery, and [1] for individual prognostic stratification [1]. Stress-induced worsening of MR—often expressed as an increase in effective regurgitant orifice area (EROA) of approximately ≥13 mm^2^—and/or a peak stress SPAP ≥ 60 mmHg identifies a higher-risk phenotype and is associated with worse outcomes [24,25]. Conversely, improvement or lack of stress may predict a better response to medical therapy [26,27]. In addition, the absence of LV contractile reserve appears to mark adverse risk in cardiomyopathies, further supporting the value of integrated stress assessment beyond MR quantification alone [28].

However, there are still several areas for improvement. During stress echocardiography, it is difficult to accurately measure the EROA or regurgitant volume during active exercise; there is a clear need to balance hemodynamic data, such as SPAP, with volumetric and contractile parameters. Furthermore, it is fundamental to identify with certainty the ‘point of no return’ for right ventricular reserve.

### 4.2. Mitral Stenosis

In mitral stenosis (MS), a marked rise in mean transmitral gradient (≈≥15 mmHg during exercise) and/or exercise pulmonary hypertension (PASP ≥ 60 mmHg); are associated with worse clinical outcomes and can suggest earlier consideration of intervention (e.g., percutaneous mitral commissurotomy in suitable anatomy) and/or closer surveillance in otherwise “asymptomatic” patients [29,30]. In asymptomatic patients with moderate MS, SE provides hemodynamically significance of MS through exercise-induced mean gradient and PAPs assessment [1,2]. In a retrospective cohort of patients with symptoms and moderate mitral stenosis, Grimaldi et al. [31] found that 76% of them (35 pts) developed exertional dyspnoea, typically in association with a stress mean transmitral gradient ≥ 15 mmHg and/or PASP > 60 mmHg. When exercise is not feasible, DSE can be performed safely [32]. At peak dobutamine dose, a mean transmitral gradient ≥ 18 mmHg identifies a high-risk subgroup and may reveal a more significant disease in up to 40% of patients otherwise considered to have moderate stenosis. During DSE SPAP assessment should not be considered.

## 5. Aortic Stenosis

### 5.1. Exercise Stress Echocardiography in Severe Aortic Stenosis Without Symptoms

In apparently asymptomatic severe AS, exercise SE is used to unmask symptoms and/or an abnormal haemodynamic response, thereby providing supportive evidence to guide timing of intervention, while also offering prognostic information [1,2]. Key risk markers include (i) a significant rise in mean aortic gradient (≈18–20 mmHg), (ii) dynamic pulmonary hypertension (e.g., SPAP > 60 mmHg at peak exercise), and (iii) an abnormal LV contractile response (no increase or a fall in LVEF) [15]. These stress-derived abnormalities have been associated with higher rates of valve-related events and adverse clinical outcomes, although their incremental value for long-term mortality remains debated [33]. In contrast, stress-related changes in global longitudinal strain (GLS) may provide additional prognostic information in selected asymptomatic patients with preserved EF [34,35]. Exercise SE is contraindicated in clearly symptomatic severe AS because of the risk of haemodynamic instability; careful clinical assessment is therefore essential before referral. The test should be stopped for limiting AS symptoms, hypotensive response, significant ST changes/arrhythmias, or new LV wall-motion abnormalities [1,2].

### 5.2. Low-Flow-Low-Gradient Aortic Stenosis

Low-flow, low-gradient (LF/LG) AS is driven by abnormal transvalvular flow conditions, ventricular dysfunction, and afterload mismatch [1,2]. Classical LF/LG AS is defined by aortic valvular area (AVA) ≤ 1.0 cm^2^ with mean gradient < 40 mmHg (and/or Vmax < 4.0 m/s) in the presence of LVEF < 50%. Low-dose DSE is used to assess LV flow (contractile) reserve (typically ≥20% stroke volume (SV) increase) and to differentiate true-severe from pseudo-severe AS by augmenting transvalvular flow. The imaging dataset should include aortic valve (AV) continuous wave (CW) doppler (Vmax, mean gradient), left ventricle outflow tract (LVOT) power wave (PW) doppler (velocity/time integral (VTI) for SV/flow), LV volumes and LVEF (±GLS where feasible), with continuous ECG and staged blood pressure monitoring [1,2]. With flow reserve, true-severe AS is suggested by a substantial rise in mean gradient with AVA remaining ≤1.0 cm^2^, whereas pseudo-severe AS is supported by an increase in AVA > 1.0 cm^2^ with only a modest gradient rise [36,37]. Although absence of flow reserve is associated with higher operative risk [38], it does not preclude LV improvement or long-term benefit after intervention; thus, management decisions should integrate the full clinical context and multimodality confirmation when needed [38]. In patients with limited flow augmentation or discordant findings, severity can be refined using the projected AVA at normal flow (AVAproj), which represents the AVA calculated at normal flow rate (250 mL/s) during low-dose dobutamine.

In the multicentre True or Pseudo Severe Aortic Stenosis (TOPAS) validation study [39,40] AVAproj outperformed conventional Doppler indices for distinguishing true-severe from pseudo-severe AS, and subsequent work suggested that AVAproj better predicts mortality than traditional DSE parameters in medically managed patients [41].

**Figure 2:**
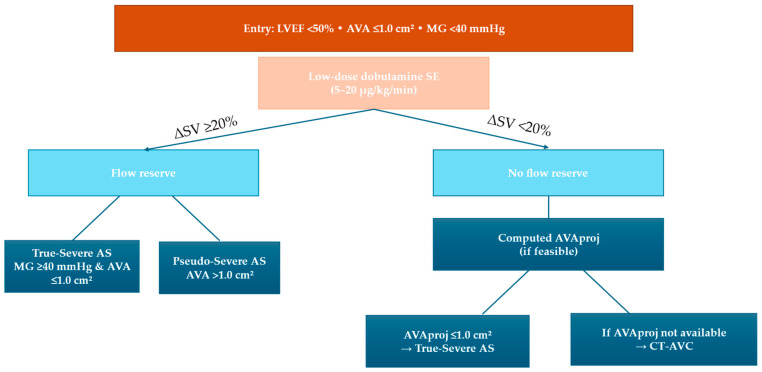
Dobutamine stress echocardiography protocol for low-flow low gradient aortic stenosis (LF-LG AS) with reduced ejection fraction. In case of absence reserve flow, the AVAproj should be integrated as shown in the panel on the right. AS: aortic stenosis; AVA: aortic valve area; AVAproj: projected aortic valve area; AVC: aortic valve calcification; CT: computed tomography; LVEF: left ventricular ejection fraction; MG: mean gradient; SV: stroke volume.

Paradoxical LF/LG AS (preserved LVEF). Paradoxical LF/LG AS (often ~25–35% of low-gradient AS) is defined by AVA < 1.0 cm^2^, mean gradient < 40 mmHg, low flow (SVI < 35 mL/m^2^), and preserved LVEF [1,2]. It is frequently associated with concentric LV remodelling, a small LV cavity, increased afterload, and/or confounding lesions (e.g., atrial fibrillation, significant MR) [42,43]. DSE may be considered, but it can be less reliable in restrictive physiology [2]; AVAproj may be preferable when feasible, and a multimodal approach—particularly CT valve calcium scoring—is often required. Outcomes are generally intermediate between classical LF/LG AS and high-gradient AS, and decisions should be Heart Team-based [44,45,46]. Figure 2 shows DSE algorithm for LF/LG AS with reduced EF, including AVAproj evaluation.

Ciampi et al. proposed an ABCDEG stress-echocardiography approach for aortic stenosis that uses SE for integrated physiological phenotyping, beyond valve “re-grading” alone [47]. The protocol combines conventional valve haemodynamics (G: transaortic gradients, LVOT flow, stroke volume, and AVA) with a structured assessment of A (stress-induced regional wall-motion abnormalities), B (exercise-related pulmonary congestion by lung B-lines), C (LV contractile reserve, including force-based indices), D (coronary microvascular function via coronary flow velocity reserve), and E (chronotropic/autonomic reserve via heart-rate reserve). This “added value” is particularly relevant in discordant phenotypes (e.g., LFLG AS) and in apparently asymptomatic severe AS, where integrating myocardial, pulmonary, microvascular and autonomic responses can refine risk stratification and better inform timing and type of intervention [47].

In conclusion, potential areas for improvement include the standardization and automation of AVAproj calculations, the use of parameters such as GLS to assess myocardial reserve, and the development of protocols that incorporate B-lines as early markers of pulmonary congestion before the onset of clinical symptoms.

## 6. Aortic Regurgitation

SE should not be used to re-grade AR severity, because stress-related tachycardia shortens diastole and may lead to underestimation of regurgitation [1,2]. However, Exercise SE offers an objective assessment of functional capacity and can provide risk stratification in asymptomatic/minimally symptomatic patients [17,18]. Observational studies suggest that absent LV reserve (e.g., ΔLVEF < 5%) is associated with subsequent LV systolic deterioration during follow-up and may correlate with postoperative LV performance, supporting stress-derived reserve as an early marker of subclinical LV dysfunction [48]. In a retrospective study including 31 patients with chronic severe AR and reduced LVEF (LVEF < 50%) who underwent aortic valve surgery, Saito et al. [49] showed that when ΔLVEF of ≥6% (with CR) during DSE was related to an improvement in post-operative LVEF with sensitivity 100%; specificity 78%; and area under the curve (AUC) 0.92. Strain- and tissue Doppler-based indices (at rest and/or during stress) are promising adjuncts for earlier detection of LV impairment, but validated cut-offs and treatment algorithms remain limited [50].

## 7. Pulmonary and Tricuspid Valve

Current consensus guidelines indicate that stress testing is a valuable tool for evaluating exercise capacity in patients with severe tricuspid regurgitation (TR) or severe pulmonary valve regurgitation/stenosis who exhibit minimal or no symptoms [3]. In this setting, supine bicycle ergometry is the preferred modality over treadmill testing; its primary advantage is the ability to perform continuous imaging, preventing the rapid normalization of parameters—such as pulmonary artery pressure—that often occurs immediately after exercise cessation.

Assessing RV functional reserve involves monitoring changes in TAPSE, RVS’, and RV fractional area change from baseline to peak stress [51]. While RV longitudinal strain is another potential marker, its reliability may diminish at heart rates exceeding 100 bpm due to constraints in temporal resolution and frame rate [52]. A blunted response in these indices signifies impaired RV reserve, a finding linked to poorer clinical outcomes in left-sided valvular pathologies.

Evidence regarding RV reserve in primary right-sided valve disease remains evolving. Notably, research on post-operative Tetralogy of Fallot (ToF) patients suggests that resting RV function can be misleading; patients with RV dilatation may demonstrate impaired reserve during stress despite seemingly normal resting TAPSE [53]. Similar studies utilizing TAPSE, fractional area change, and longitudinal strain have identified latent RV dysfunction in ToF patients with residual pulmonary regurgitation [53]. Ultimately, further research into occult RV dysfunction is essential to optimize surgical timing—distinguishing between patients who require earlier intervention and those whose irreversible damage may render surgery excessively risky.

## 8. Post Operative Assessment

After valve repair or replacement, stress echocardiography is performed to investigate exertional symptoms or discordant/borderline resting doppler data by distinguishing physiological prosthetic haemodynamics from clinically relevant obstruction or patient–prosthesis mismatch (PPM) [37]. The exam should document symptoms/workload, blood pressure/ECG, and the flow–gradient relationship (heart rate and stroke volume surrogates) alongside peak-stress Doppler gradients; exercise is preferred when feasible, whereas low-dose dobutamine is useful in low-flow states or when exercise capacity is limited [1]. An abnormal haemodynamic response is suggested by a disproportionate stress-related rise in mean transprosthetic gradient (commonly >20 mmHg for aortic prostheses and >10–12 mmHg for mitral prostheses), particularly when accompanied by stress pulmonary hypertension; conversely, a modest gradient rise in the setting of robust flow augmentation may indicate predominantly flow-mediated changes rather than fixed obstruction [54]. When stress findings suggest prosthetic dysfunction (thrombus/pannus, structural degeneration) or clinically important PPM, results should be integrated by multimodality imaging (e.g., TEE and/or CT) [55].

In Table 2, the indications, stress protocols, and recommended parameters for stress echocardiography across VHD phenotypes are indicated.

## 9. Future Perspectives

The future of SE in the management of VHD is moving toward a more comprehensive, “multiparametric” approach that transcends simple valve re-grading. While traditional parameters remain foundational, the integration of advanced technologies such as 3D echocardiography and speckle tracking (specifically GLS) is expected to further refine our ability to detect subclinical myocardial impairment and predict adverse outcomes.

Future research is increasingly focused on integrated physiological phenotyping, exemplified by the “ABCDEG” protocol. This holistic framework aims to combine standard valvular hemodynamics with assessments of pulmonary congestion via lung B-lines, coronary microvascular function, and autonomic heart-rate reserve. By unmasking the complex interplay between the valve, the ventricles, and the pulmonary circulation, these evolving techniques will provide the Heart Team with a more robust decision-making tool. Ultimately, these advancements seek to optimize the timing of surgical or transcatheter interventions, ensuring that treatment is tailored to the individual’s dynamic hemodynamic profile rather than resting data alone.

Furthermore, the application of artificial intelligence (AI) protocols could, on the one hand, reduce inter- and intra-operator variability and could facilitate the creation of diagnostic pathways that include the development of markers that could reduce the impact of SE in the diagnostic workup of VHD [56].

## 10. Conclusions

In conclusion, SE has evolved into an indispensable tool in the modern management of VHD. By providing a dynamic assessment that resting imaging cannot capture, it successfully unmasks the true severity of valvular lesions and identifies high-risk markers such as exercise-induced pulmonary hypertension and blunted contractile reserve. These insights are crucial for guiding the timing of surgical or transcatheter interventions, particularly in patients where resting data and clinical symptoms are discordant. Looking ahead, the integration of novel markers like GLS and 3D echocardiography, particularly when combined with AI, may lead to the development of advanced clinical protocols.

## Figures and Tables

**Table 1:** The recommended stress modality, minimum dataset, high-risk stress findings, and typical clinical implications across MR phenotypes.

Clinical Scenario	Preferred Stress	Minimum Dataset	High-Risk Stress Findings	Typical Implication
Primary MR, severe at rest, “asymptomatic”/equivocal	Exercise (semi-supine bicycle preferred)	Symptoms/workload; BP/ECG; SPAP; LV reserve (ΔLVEF/longitudinal response); RV response when feasible	Symptoms; SPAP ≥ 60 mmHg; absent LV reserve; exercise-induced RV dysfunction	Earlier valve-centre referral/closer follow-up; supports timing decisions when uncertain
Primary MR mild–moderate at rest with disproportionate dyspnoea	Exercise	MR quantification (VC/PISA if feasible); SPAP; LV function/reserve; symptoms	MR worsening ≥ 1 grade; rising SPAP; limited reserve with objective limitation	Clarifies symptom attribution and “dynamic burden”; intensify surveillance/management
Secondary MR with suspected dynamic worsening	Exercise	Stress MR quantification (EROA/RegVol if feasible); SPAP; LV geometry/function; flow reserve response	ΔEROA ≥ 13 mm^2^ and/or major regurgitant increase; SPAP ≥ 60 mmHg; poor flow reserve response	Identifies high-risk dynamic MR → optimise HF therapy/device strategy; heart-team discussion
Ischaemic MR/LV dysfunction, recurrent pulmonary oedema or unclear mechanism	Exercise (or selected pharmacologic if unable)	MR dynamics; SPAP; LV reserve; consider ischaemia mechanism	Marked stress MR increase consistent with trigger physiology	Integrated strategy (revascularization ± MR approach)

BP: blood pressure; ECG: electrocardiogram; EROA: effective regurgitant orifice area; HF: heart failure; LV: left ventricle; LVEF: left ventricle ejection fraction; MR: mitral regurgitation; PISA: proximal isovelocity surface area; RegVol: regurgitation volume; SPAP: systolic pulmonary artery pressure; VC: vena cava. Blues is the title of the column; sky blues is the explanation.

**Table 2:** Indication, stress protocols, and recommended parameters for stress echocardiography across VHD phenotypes.

Valvular Disease	Indication	Stress Protocol	Recommended Parameters
AS	Classical LFLG severe AS	Dobutamine	Mean PG, FR, SV, and LV systolic reserve; calculate AVAproj
AS	Asymptomatic severe AS	Exercise	Symptoms/workload, mean PG, AVA, and exercise-induced PH (Ex-PH)
AS	Equivocal symptomatic moderate AS	Exercise	Adjuncts may help: Zva, GLS, and PR during exercise
MR	Primary MR	Exercise	Symptoms/workload; MR increase; Ex-PH; consider LV (LVEF/GLS) and RV (TAPSE) reserve
MR	Secondary MR	Exercise	Symptoms/workload; MR increase; Ex-PH
MS	Rheumatic MS	Exercise	Increase in mean transmitral PG and Ex-PH
Post-operative	Suspected PPM/prosthesis degeneration	Exercise or dobutamine	Transprosthetic PG, EOA, DVI, and LV systolic reserve
Post-operative	Mitral annuloplasty	Exercise or dobutamine	Transmitral PG and Ex-PH
Others	AR	Exercise	Not established; LV reserve/GLS, ESV, and TAPSE may be useful

AR: aortic regurgitation; AS: aortic stenosis; AVA: aortic valve area; AVAproj: projected aortic valve area; DVI: Doppler velocity index; EOA: effective orifice area; ESV: end-systolic volume; Ex-PH: exercise-induced pulmonary hypertension; FR: flow reserve; GLS: global longitudinal strain; LFLG: low-flow low-gradient; LV: left ventricle; LVEF: left ventricular ejection fraction; MR: mitral regurgitation; MS: mitral stenosis; PG: pressure gradient; PPM: patient–prosthesis mismatch; PR: peak response; RV: right ventricle; SV: stroke volume; TAPSE: tricuspid annular plane systolic excursion; Zva: valvulo-arterial impedance. Blues is the title of the column; sky blues is the explanation.

## Data Availability

No new data were created or analyzed in this study. Data sharing is not applicable to this article.
